# Interference Mitigation in Automotive Radars Using Pseudo-Random Cyclic Orthogonal Sequences

**DOI:** 10.3390/s19204459

**Published:** 2019-10-15

**Authors:** Sruthy Skaria, Akram Al-Hourani, Robin J. Evans, Kandeepan Sithamparanathan, Udaya Parampalli

**Affiliations:** 1 School of Engineering, RMIT University, Melbourne VIC 3000, Australia; akram.hourani@rmit.edu.au (A.A.-H.); kandeepan.sithamparanathan@rmit.edu.au (K.S.); 2 Department of Electrical and Electronic Engineering, The University of Melbourne, Melbourne VIC 3010, Australia; robinje@unimelb.edu.au (R.J.E.); udaya@unimelb.edu.au (U.P.)

**Keywords:** automotive radar, stepped frequency radar, interference mitigation, sequence design

## Abstract

The number of small sophisticated wireless sensors which share the electromagnetic spectrum is expected to grow rapidly over the next decade and interference between these sensors is anticipated to become a major challenge. In this paper we study the interference mechanisms in one such sensor, automotive radars, where our results are directly applicable to a range of other sensor situations. In particular, we study the impact of radar waveform design and the associated receiver processing on the statistics of radar–radar interference and its effects on sensing performance. We propose a novel interference mitigation approach based on pseudo-random cyclic orthogonal sequences (PRCOS), which enable sensors to rapidly learn the interference environment and avoid using frequency overlapping waveforms, which in turn results in a significant interference mitigation with analytically tractable statistical characterization. The performance of our new approach is benchmarked against the popular random stepped frequency waveform sequences (RSFWS), where both simulation and analytic results show considerable interference reduction. Furthermore, we perform experimental measurements on commercially available automotive radars to verify the proposed model and framework.

## 1. Introduction

The rapidly increasing deployment of small, low cost wireless based remote sensing devices is leading to an increasing concern about spectrum crowding and device interference. A prominent example is the small scale radar used for vehicle safety and autonomy, for UAV navigation, for collision avoidance and mapping and for hand gesture recognition [1], where interference could seriously jeopardize the utility of these systems. In this paper, we focus on interference mitigation for automotive radars; however, our results are equally applicable to UAV systems and more broadly to a diverse range of Internet-of-things applications.

Currently automotive radars are limited to high-end vehicles due to development and integration costs. However, very low-cost consumer radar technology is imminent [2,3,4,5] and the global market volume of automotive radars is expected to experience a compound annual growth rate of 22.5% over the period of 2017–2023 [6]. Many spectrum management authorities have already regulated the spectrum bands around 24 GHz and 77 GHz [7] primarily for shared use by automotive radars. This sharing of the spectrum will result in overlapping frequency bands with inevitable adjacent channel interference [5].

Interference in automotive radar systems can arise from vehicles traveling in opposite directions, vehicles traveling in the same direction fitted with forward-looking and backward-looking radars and vehicles at intersections [8]. The interference in all of these cases is caused by the use of shared spectrum and the lack of coordination between radars due to the native absence of a centralized control and resource allocation mechanism. Interfering signals can cause severe problems for radars; for example, a strong signal transmitted from one radar can saturate the receiver of another radar resulting in complete loss of functionality. Even small signals can deteriorate the performance of the victim radar by increasing the noise floor of the receiver [9], which in turn reduces the sensitivity of the system. Thus, it directly affects the detection performance, which in turn impacts the autonomy and safety systems. It is therefore critically important to understand mutual interference in terms of spatial, temporal and spectral parameters and devise practical schemes to mitigate its negative effects.

In this paper, we propose a novel technique to mitigate multi-radar interference in an automotive radar context by designing an adaptive family of radar waveform sequences which are easily learnable by other radars. These sequences always maintain a minimum frequency separation (or a frequency gap) between a radar’s instantaneous transmitting frequencies. Thus, forming a frequency guard which guarantees orthogonality between sequences. Using this scheme, the available spectrum can be efficiently shared among radars in a fully decentralized manner, avoiding the need for a centralized control system. Thus, radars can adaptively manage frequency separation according to the current interference environment.

The main contributions of this paper are:

- Development of a mathematical model for the mutual interference in automotive radar systems.
- Development of an analytic framework to characterize the effective interfering power parametrized according to the frequency separation between radars. This framework is applicable to a large class of radar waveforms.
- Representation of a new family of waveform sequences which are capable of mitigating the effects of multi-radar interference without the need for any centralized co-ordination.
- Development of a new statistical characterization of mutual interference in an analytically tractable form.

## 2. Brief Review of Existing Work on Automotive Radar Interference

There are many projects around the world aimed at developing practical mitigation techniques for automotive radar interference. For example, the project MOSARIM [10] is one of the first industry-led attempts to explore the effects of mutual interference in a comprehensive manner involving experimental road measurements and complex ray-tracing simulations. Certain possible interference mitigation techniques are also suggested by this project.

The interference between radars which use FMCW waveform is investigated in [9,11], where it is characterized according to the waveform parameters such as sweep time, phase shift, etc. These studies show that the behaviour of the interference significantly depends on the spectral and temporal characteristics of the waveform. Ghost targets due to interference were studied analytically in [12] and found to occur when two identical radars have identical waveforms. A comparative study of interference effects using FMCW against random pulse sequences is studied in [13], which shows that the impact is relatively similar. Automotive radar interference is not only affected by the radar waveform, but also by the road geometry and traffic density. The work in [14] provides a statistical study of the power level of interference with different road geometry and traffic conditions. The first attempt to model the stochastic behaviour of automotive radar interference based on the spatial randomness in the position of the interferers is provided in [8].

Interference mitigation in automotive radars could be achieved by various techniques using different antenna polarization [10,15,16], separate time slots [17], separate radio channels [18], signal processing algorithms [19,20,21], antenna nulling methods [22] and coding techniques [23]. Table 1 provides the list of works on interference mitigation in automotive radars according to the applied technique.

In order to reduce the received power from the direction of interferer, the concept of digital beam forming is applied in [22,24]. Another study adapts frequency hoping inspired by European bats (European bats uses frequency hopping to avoid interference between each other) in [25] to avoid collision of the same frequencies, by adaptively jumping to either side of the center frequency of the chirp. Similar work is done in [3], where a 77 GHz radar transceiver is implemented with randomly changing center frequencies of the chirp. Signal processing techniques like the maximally stable extremal regions algorithm are adopted in [26] to effectively estimate and cancel the mutual interference. Interference mitigation using pseudo-random sequences is studied in [15,17,27], which shows considerable elimination of mutual interference in automotive scenarios. Practical system algorithms that efficiently implement random frequency stepping in automotive applications have been filed by our team in these two patents [28,29] suggesting a reduced interference when utilizing this scheme. However, most of these implementations are investigated using either parametric simulations [9,12,13,21] and/or experimentation [3,11,24] without providing the ability to optimize the controlling parameters based on certain performance criteria. In this paper, the main differentiator is that it provides an explicit analytic relation between the performance criteria and the operational parameters.

## 3. Interference Modelling

Future vehicles with advanced driver assistance are likely to be equipped with 8–10 radars which will give an overall 360${}^{\circ }$ coverage [13] to scan the environment. According to the International Telecommunication Union (ITU), automotive radars can be categorized as [8],

- Short range radars (SRR) for distances of 0.15–30 m
- Medium range radars (MRR) for distances of 1–100 m
- Long range radars (LRR) for distances of 10–250 m

SRR/MRRs are typically used for blind spot detection, lane changing assistance, parking assistance, side impact and self-parking, while LRRs are used for automatic cruise control, collision avoidance, cross traffic alert, etc. LRRs are more vulnerable to interference because of the long range, where typically such radars have narrow beam-width antennas for detecting far objects.

**Figure 1:**
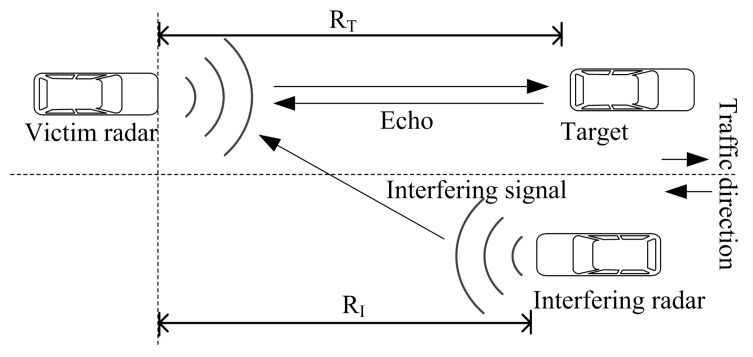
A simplified radar interference model with two lanes in opposite directions.

The main cause of interference is vehicles traveling in the opposite direction equipped with LRRs operating in the same spectral band. A simplified typical automotive radar interference scenario is shown in Figure 1, where a victim radar suffers interference from vehicles traveling in the opposite direction and falling within a certain range which depends on the transmit and receive antenna beam-widths among other things.

Referring to Figure 1 the received power at the radar from the target has a well-known relation given as follows [30],

(1) $$\begin{array}{cc} \;P_{R} & =\underset{\mathrm{Incident}\;\mathrm{power}}{\underset{︸}{\frac{P_{o}G}{4\pi R_{T}^{2}}}}\times \underset{\mathrm{Reflected}\;\mathrm{power}}{\underset{︸}{\frac{\sigma_{c}A_{e}}{4\pi R_{T}^{2}}}}=\frac{P_{o}G^{2}\sigma_{c}\lambda^{2}}{{\left(4\pi \right)}^{3}R_{T}^{4}}, \end{array}$$

where $A_{e}=\frac{G\lambda^{2}}{4\pi }$, is the effective aperture of the antenna, $P_{R}$ is the reflected power form the target, $P_{o}$ is the transmitted power, *G* is the gain of the radar’s antenna, λ is the wavelength of the signal, $\sigma_{c}$ is the radar cross-section (RCS), and $R_{T}$ is the distance between the transmitter and the target. Likewise, the power received at the radar from the interferer, considering free space propagation, is given by the Friis transmission equation [11] as,

(2) $$\;P_{I}=\frac{P_{o}G^{2}\lambda^{2}}{{\left(4\pi R_{I}\right)}^{2}}\;,$$

where $P_{I}$ is the received power at the victim, $P_{o}$ is the transmitted power from the interferer, assuming that the transmitted power from the interfering and the victim radar are equal, also the interferer and the victim have the same system gain and $R_{I}$ is the distance from the interfering radar to the victim radar.

From Equations (1) and (2) it is clear that the target echo will have far less power than the interfering signal by a factor of at-least $1/R_{T}^{2}$ (when the target and the interferer are at the same distance). Moreover, as indicated above, the front-end of the victim radar can become saturated by the interferer transmit signal when the interferer passes the victim causing it to be temporarily blinded. The separation point at which this happens depends upon several factors including the victim low-noise amplifier dynamic range, the input bandpass filters and the interferer transmit power.

Assuming that the victim receiver is not blinded then depending on the particular characteristic of the victim radar receiver there will be an effective interfering power that makes its way to the intermediate frequency (IF)/baseband section of the receiver. We define this fraction of total interfering power as $\zeta_{o}$∈[0,1]. This $\zeta_{o}$ depends on the bandwidth of the IF/baseband, the bandwidth of interfering signal and the local oscillator stability. Accordingly, the signal-to-interference-plus-noise ratio (SINR), is given by,

(3) $$\begin{array}{c} \mathrm{SIN}R=\frac{P_{R}}{P_{I}\zeta_{o}+\sigma_{n}^{2}}, \end{array}$$

where $\sigma_{n}^{2}$ is the noise power at the receiver.

Spectrum regulators impose certain restrictions on the maximum allowed transmitting power (the regulations are usually related to the maximum equivalent isotropic radiated power (EIRP)) [7] and the utilized frequency band. Thus far there are no regulations on the choice of transmit waveforms that can be used by automotive radars [13]. However, the interference behaviour depends on the transmit waveform as it can be seen in the example of linear FM, where it results in ghost targets (when the radars are synchronized) [12] and noise floor level is increased [9]. Thus, by carefully designing the waveforms, a worthwhile reduction in interference can be achieved.

### Radar Architecture

**Figure 2:**
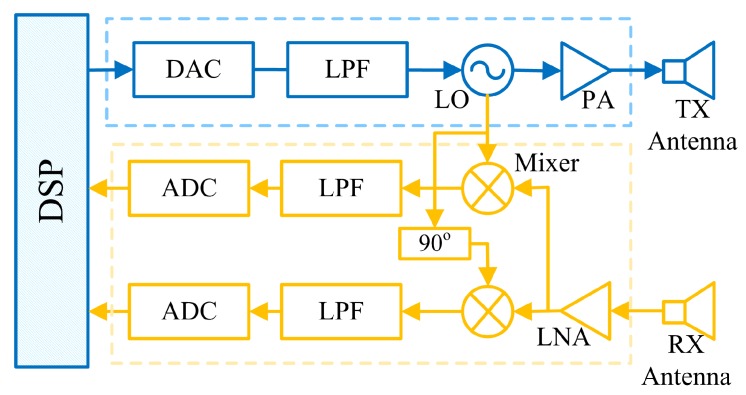
A high level block diagram of a next generation [5,31,32] radar system.

In order to characterize the interference, it is important to understand the architecture of next generation radar front-end [5]. As depicted in Figure 2, a generic radar front-end consists of a power amplifier (PA) connected to the transmit (TX) antenna, while a separate receive (RX) antenna is connected to a low-noise amplifier (LNA).

A digital signal processing (DSP) unit drives a digital-to-analog converter (DAC) that in turn controls the frequency of a voltage controlled oscillator (VCO). Thus, the frequency can be stepped, or swept, according to the desired transmit waveform. The receive signal, on the other hand, is coherently down-converted at the mixer and low pass filter (LPF) using the same frequency produced by the VCO into an in-phase (I) and quadrature (Q) baseband component which are sampled by two analog-to-digital converters (ADC) and then fed to the DSP.

In the context of interference, the performance of the VCO is very important because the oscillator phase noise spreads the signal (and interference) energy across adjacent frequencies. Moreover, while each radar can have an accurate crystal locked oscillator there is no radar–radar frequency or phase synchronization, so individual radar oscillators effectively drift with respect to each other. Thus, there is a high chance of interfering signals even if the interferer is not at exactly the same frequency as the victim radar operating frequency. If the interfering signal passes through the IF/baseband filters in the receiver, it will degrade the performance of the victim radar.

## 4. Pseudo-Random Cyclic Orthogonal Sequences

**Figure 3:**
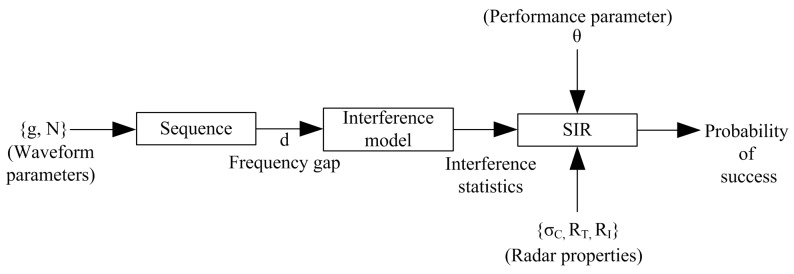
A block diagram of the proposed analytic framework for characterizing automotive radar interference.

In order to mitigate the mutual interference in automotive radars, it is important first to study the interference characteristics and behaviour. Figure 3 shows the proposed analytic framework for characterizing the interference. Our aim is to quantify the interference performance of the automotive radar based on the statistical characteristics of the waveform. The framework starts with the generation of the proposed new waveform sequence. This leads to the computation of the interference model parametrized according to the frequency separation *d* between the victim and interfering radars. With this model we calculate the statistical parameters to characterize the performance of the new transmit waveform in terms of signal-to-interference ratio (SIR).

The last box in Figure 3 deals with the prediction of the probability of success, i.e., the probability of the SIR exceeding a given threshold θ, which can be determined using the waveform parameters and the physical situation such as the target distance, interferer distance, RCS, etc. The performance metric is expressed in terms of the signal-to-interference ratio (SIR), while it is crucial to note that there is a direct monotonic relationship between the SIR and the detection probability of a radar [33], such that a better SIR implies better detectability. However, the detectability and separation of targets is also dependent on other parameters such as radar sensitivity, RCS, signal strength, waveform design and on the type of detector.

To design a waveform with tractable properties we use the concept of pseudo-random stepped frequency hopping. Practical advantages of using random stepped frequency waveforms in automotive radar applications often refer to its high SIR due to its narrow instantaneous bandwidth at each tone [31]. The waveform parameters and the range–Doppler extraction of the proposed sequence is same as that of the random-stepped frequency waveform. We divide the available bandwidth into equally spaced frequency tones. The tones are pseudo randomly ordered in a particular manner, such that the users maintain a certain minimum frequency separation (gap) when concurrently accessing the spectrum.

### 4.1. Random Stepped Frequency Radar

In a random stepped frequency waveform [34], the available bandwidth is divided into equally spaced frequency tones separated by Δf=BW/N, where the allowed frequency pool is represented as $\left\{f_{1},\cdots ,f_{n},\cdots f_{N}\right\}$, where BW is the allocated bandwidth and *N* is the number of frequency tones. The frequency tones are randomly selected without replacement from the pool of allowed frequencies and transmitted as a pulse train. This pulse train where each pulse has a different frequency (tone) is equivalent to a narrow synthetic pulse when processed using the inverse Fourier transform (IDFT) [35] to provide range and velocity information of a target. A detailed discussion on the signal processing of random stepped frequency waveform for a target’s distance and velocity estimation is given in [5,35,36,37,38], while the angle of arrival estimation made possible with the use of multiple receivers is given in [39].

The randomization in the sequence of tones can significantly reduce the interference compared to its linear version [34], which is the linear stepped frequency waveform. Moreover, this random stepping in frequency tones helps to avoid range-velocity coupling problems that are present in linear stepped-frequency waveforms [40].

The design of a proper waveform sequence or a family of sequences can significantly mitigate the interference arising from a large number of radars operating in close spatial and spectral proximity. We introduce a new family of sequences called pseudo random cyclic orthogonal sequences (PRCOS) which are capable of adaptively reducing the effects of mutual interference in dense multiple radar situations such as those found in automotive radars or UAV swarms. The proposed PRCOS is peculiar due to its generation and each sequence shared among the radars are cyclic shifted versions of each other. By orthogonal we mean that the transmitting frequencies of the radars will always maintain a minimum frequency gap between the radars, which aids in limiting the effective interfering power. The PRCOS are easy to learn, thus enabling a radar to dynamically learn the sequence being used by another radar and consequently avoid spectral collision. The sequence is pre-shared among the radars, such that each radar entering the new environment listens to the other radars and learns (from the beat frequency) which frequency tones others are using. The proposed sequence is constructed under the following assumptions:

- All available frequencies need to be used within the pulse train of length *N*.
- The sequence does not have repeated elements within a pulse train, since it is sufficient to probe the scene once at each frequency tone.
- The sequence is cyclic, thus it repeats itself periodically after *N* pulses.

In general, a unique sequence, i.e., with non-repeated elements, is taken from the set S∈$\left\{S_{1},S_{2},\cdots ,S_{k}\right\}$ which is permutations ${}_{N}P_{k}\left[S_{o}\right]$ of the ordered set,

(4) $$S_{o}=\left\{f_{1},\cdots ,f_{n},\cdots f_{N}\right\}.$$

Suppose a single unique cyclic sequence $S_{i}$ is shared among all radars. Then it is sufficient to listen to one tone duration only to learn the current phases of all other radars. Defining the phase ϕ as the relative starting point of the sequence $S_{i}$ with respect to the listening radar and after learning the utilized phases, a radar will have a choice to pick up any available orthogonal phase to avoid spectral collision with other radars.

### 4.2. Generating the PRCOS Sequence

**Figure 4:**
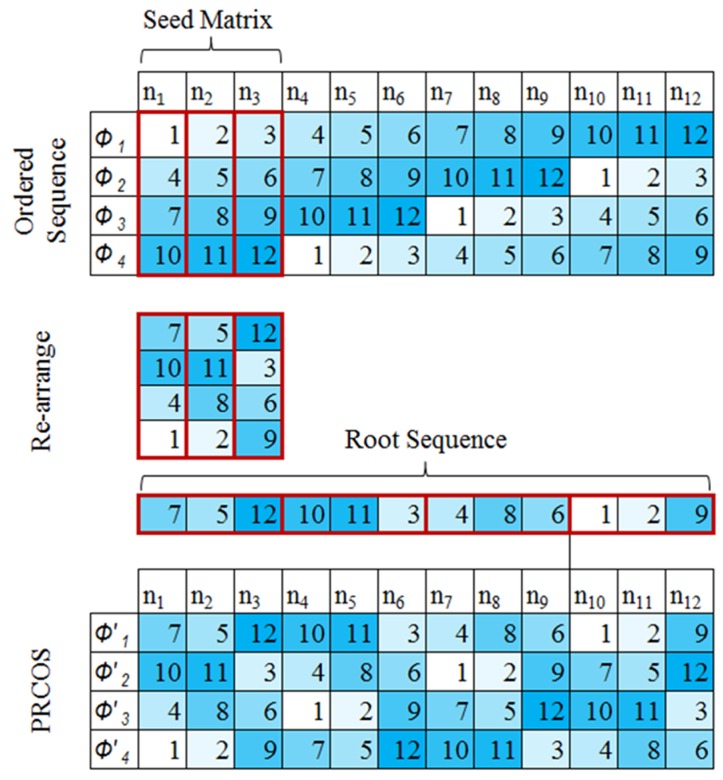
A short sequence example of the proposed generating algorithm with *N* = 12, *g* = 3 and *M* = 4.

Since all sequences are permutations of the ordered set, we start from $S_{o}=\left\{f_{1},\cdots ,f_{n},\cdots f_{N}\right\}$ with *N* elements. Suppose there exists a certain frequency guard, where two tones used by two different radars should be spaced by more than this frequency guard *g*, then the permitted phases $\Phi =\{\phi_{1},\cdots ,\phi_{m},\cdots \phi_{M}\}$ of the cyclic sequence are,

$$\begin{array}{cc} \; & \phi_{1}=\left\{f_{1},.,\;\;\;\;\;\;\;\;\;\;\;\;\;\;\;\;f_{n},.,\;\;\;\;\;\;\;\;\;\;\;\;\;\;\;\;\;\;\;\;\;\;\;\;\;f_{N}\right\},\\ \; & \phi_{2}=\left\{f_{1+g},.,\;\;\;\;\;\;\;\;\;\;\;\;\;f_{(n+g-1)(\mathrm{mod}N)+1},\ldots \;\;\;\;\;\;\;\;\;\;\;\;\right\},\\ \; & \vdots \\ \; & \phi_{m}=\left\{f_{1+(m-1)g},.,\;\;\;\;\;\;f_{(n+(m-1)g-1)(\mathrm{mod}N)+1},\ldots \;\;\;\;\;\;\right\},\\ \; & \vdots \\ \; & \phi_{M}=\left\{f_{1+(M-1)g},.,\;\;\;\;\;f_{(n+(M-1)g-1)(\mathrm{mod}N)+1},\ldots \;\;\;\;\;\;\right\}, \end{array}$$

where *M* is the number of non-overlapping sequences calculated as $M=\left\lfloor \frac{N}{g}\right\rfloor$. If the ratio $\frac{N}{g}$ is an integer, then the first *g* columns of tones of the permitted phases can reconstruct the full sequence, where $f_{(g+mg)(\mathrm{mod}\;N)+1}=f_{\left(1+(m+1)g)(\mathrm{mod}\;N\right)}$, noting that m∈[1,M] and n∈[1,N] represent the rows and columns, respectively. An illustrative example is shown in Figure 4 for a short sequence of N=12 tones and frequency guard g=3. It is clear from this example that the allowed permutations of the ordered set can only occur by interchanging the elements separated by cyclic distance kg,k∈[1,M−1], any other permutation will cause a collision with other phases.

We also note from the example that the elements in the columns separated by a cyclic distance kg,k∈[1,M−1] are in fact vertically rotated versions of each other. Therefore, if the first *g* columns are permuted vertically, then *M* orthogonal (none-overlapping) sequences can be generated. A summary of the steps required to generate this kind of PRCOS is:

- Generate the seed matrix $X_{M\times g}$ matrix with elements $x_{m,n}=n+\left(m-1\right)g$, n∈[1,N], m∈[1,M].
- Permute the columns so that the resulting matrix $Y_{M\times g}=P\left[X,1\right]$ with elements $y_{m,n}$, where P[.,1] means column-wise permutation.
- Re-order the matrix in a vector, $Z_{1\times gM}$ such that its elements $z_{1,g(m-1)+n}=y_{m,n}$,

where the vector $Z_{1\times N}$ is the generated PRCOS.

The seed matrix has *g* columns and *M* rows, where each column is randomly permuted M! ways. Therefore *g* columns permute M! which gives ${\left(M!\right)}^{g}$ possible arrangements for the root sequence. Only one root sequence is selected and is pre-shared among all radars. Once the root sequence is fixed, the remaining M−1 sequences can be obtained by the cyclic shifting of the above generated root sequence by kg,k∈[1,M−1].

## 5. Performance Analysis of PRCOS

In order to evaluate the interference performance of the proposed PRCOS, the metric we adopt is in terms of signal-to-interference ratio. It is also important to note that there is a direct monotonic relationship between the SIR and the detection probability of a radar [33], such that a better SIR implies better detectability. The amount of interfering signal (in turn SIR) depends on the factors such as the frequency separation between the radars, receiver characteristics like VCO performance and LPF bandwidth. Therefore, it is critical to study the VCO phase noise to characterise the interfering signal in terms of the frequency separation between the radars and the LPF bandwidth.

### 5.1. Oscillator Phase Noise

The spectrum of an ideal oscillator is an impulse at the generated frequency, but due to the effects of phase noise the spectrum of a practical oscillator is broadened. Phase noise in oscillators can be modelled as random fluctuations in phase over time, which results in the spreading of energy to adjacent frequencies [41].

**Figure 5:**
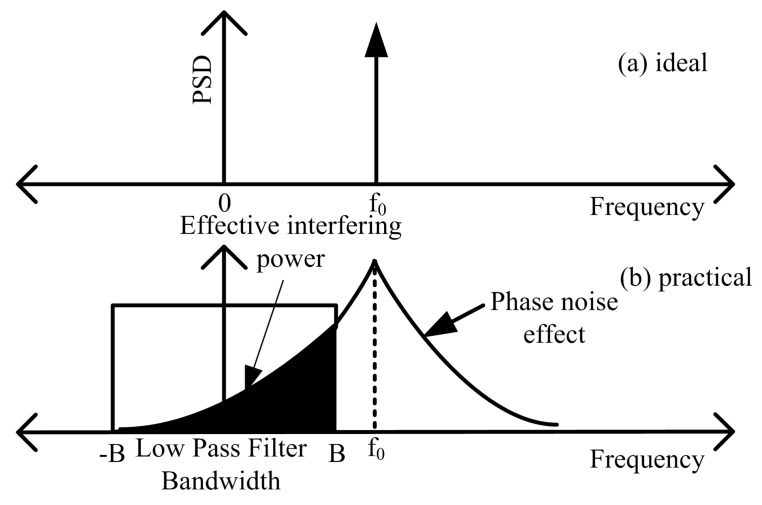
(**a**) Spectrum of an ideal oscillator; (**b**) spectrum of a practical oscillator.

Phase noise can be seen as the combination of white Gaussian noise and coloured noise [42], where the coloured noise is caused by the low frequency flicker noise which is dominant at small offset frequencies, while the white Gaussian noise is contributed by the thermal processes in the receiver electronics and is more significant at higher offset frequencies. In order to visualize the difference between the ideal and practical spectrum of an oscillator we provide an illustration in Figure 5.

One of the widely used models for phase noise is the Lorentzian spectrum [43]. By ignoring the flicker noise a typical Lorentzian power spectral density (PSD) can be represented as follows [43],

(5) $${£}_{\mathrm{dBc}/\mathrm{Hz}}\left(f\right)=10\log_{10}\left(\frac{{f_{o}}^{2}\alpha }{\pi^{2}{f_{o}}^{4}\alpha^{2}+f^{2}}\right)\;\;,$$

where $f_{o}$ is the center frequency of the oscillator, α is a scalar constant that defines the phase noise level in the oscillator, *f* is the frequency offset from the center frequency.

For short range radars the effect of phase noise is significantly mitigated by the range correlation effect [44] because in practical radars the transmitter and receiver share the same oscillator, but for radar-to-radar transmissions phase noise is not correlated because individual local oscillators are not synchronized. If the IF/baseband bandwidth is ±B then it is clear that the level of interfering power depends on both the VCO performance and IF/baseband filters, which determine the factor $\zeta_{o}$ introduced above.

### 5.2. Effect of Transmit Waveform

The model in Section 5.1 is for an unmodulated carrier waveform; however, in practice, the carrier frequency is usually modulated by periodic pulse waveforms, such as a short rectangular pulse, a linear frequency, stepped frequency modulation, etc. By considering the modulator as a linear-time invariant system with an impulse response g(t) and a corresponding transfer function G(f)., accordingly the output PSD $S_{YY}\left(f\right)$ is given by,

(6) $$\begin{array}{c} S_{YY}\left(f\right)={\left|G\left(f\right)\right|}^{2}\times S_{XX}\left(f\right)\;, \end{array}$$

where $S_{XX}\left(f\right)$ is the input PSD [45].

In the case of a square pulse the power transfer function is given by,

(7) $$\begin{array}{c} {\left|G\left(f\right)\right|}^{2}=T{\sin c}^{2}\left(\pi Tf\right)\;, \end{array}$$

where *T* is the pulse width. Therefore the normalized PSD at the output of the pulse modulator is given as,

(8) $$\begin{array}{c} {£}_{mod}\left(f\right)=G_{o}T{\sin c}^{2}\left(\pi Tf\right)\times £\left(f\right)\;, \end{array}$$

where $G_{o}=\frac{2f_{o}^{2}\pi^{4}T\alpha }{2\pi^{2}T-\sqrt{\frac{1}{f_{o}^{4}\alpha^{2}}}+\sqrt{\frac{1}{f_{o}^{4}\alpha^{2}}}\cos h\left(2f_{o}^{2}\pi^{2}T\alpha \right)-\frac{\sin h(2f_{o}^{2}\pi^{2}T\alpha )}{f_{o}^{2}\alpha }}$ is the normalizing factor.

### 5.3. Pulse Waveform and Phase Noise

The spectral characteristics of a random stepped frequency waveform are studied by considering an individual rectangular pulse of width *T* and amplitude *E* represented as,

(9) $$\;E\;{\mathrm{rect}}_{T}\left(t\right)\overset{F}{\underset{F^{-1}}{⇄}}ET\sin c\left(Tf\right),$$

When this pulse is modulating an ideal carrier of frequency $f_{o}$ the resulting spectrum is a sinc function centered at $f_{o}$ that is,

(10) $$\;E\;{\mathrm{rect}}_{T}\left(t\right)\exp\left(j2\pi f_{o}t\right)\overset{F}{\underset{F^{-1}}{⇄}}ET\sin c\left(T\left(f-f_{o}\right)\right).$$

Therefore, the overall frequency spectrum of a stepped frequency waveform can be seen to be an overlap of multiple sinc functions centered at each tone $f_{n}$. As mentioned in Section 5.2; VCO phase noise also contributes to the shape of the spectrum. As previously indicated (refer to Figure 5), only a fraction of the total interfering power enters the receiver’s IF/baseband stage depending on the instantaneous frequency separation between the victim radar and the interfering radar.

### 5.4. Effective Interfering Power

In this section, we explore the statistical distribution of the distances between two tones based on the suggested PRCOS. Assuming two radars randomly select two tones $f_{i}$ and $f_{j}$ such that i≠j, we obtain the effective normalized interfering power that makes its way through the IF (or baseband) filter of the victim radar as follows:

(11) $$\begin{array}{cc} \zeta_{o}\left(d\right) & =\int_{-\infty }^{\infty }\left|H{\left(f\right)}^{2}\right|\times {£}_{\mathrm{mod}}\left(f-d\right)df, \end{array}$$

where H(f) is the frequency response of the IF (or baseband) filter. For simplicity, we consider an ideal LPF with a transfer function H(f) = 1 in the range ±B. The effective normalized interfering power in Equation (11) does not have a closed-form expression, therefore we propose a semi-analytical approach for expressing $\zeta_{o}$ in a closed-form. We develop a simple and explicit empirical model which fits well with both experimental data (indicated in Section 7) and the analytic Lorentzian model given in Equation (11). This empirical model is formed such that the effective normalized interfering power can be approximated using a generic sigmoid function with the form $S\left(x\right)=\frac{e^{x}}{1+e^{x}}$ with $\frac{dS(x)}{dx}=\frac{e^{x}}{{\left(1+e^{x}\right)}^{2}}$.

Therefore, the proposed empirical model is given by,

(12) $$\begin{array}{cc} \zeta_{o}\left(d\right) & =\int_{-B}^{B}\frac{A\exp\left((f-d)/C\right)}{{\left(1+\exp((f-d)/C\right))}^{2}}df\\ \; & =\frac{AC\sinh\left(B/C\right)}{\cosh\left(B/C\right)+\cosh\left(d/C\right)}, \end{array}$$

where *A* and *C* are empirical parameters, *A* is proportional to the power of the signal and *C* represents the spread of the signal. The expression of Equation (12) is given in terms of sinh and cosh instead of the exponential function because of the finite interval integration.

The actual SIR resulting from a single interfere is given by,

(13) $$\begin{array}{cc} \gamma & =\frac{P_{R}}{P_{I}\zeta_{o}}=\frac{P_{o}G^{2}\left(\frac{\sigma_{c}\lambda }{{\left(4\pi \right)}^{3}R_{T}^{4}}\right)}{P_{o}G^{2}\left(\frac{\lambda }{{\left(4\pi \right)}^{2}R_{I}^{2}}\right)\zeta_{o}}\;,\\ \; & =\frac{\sigma_{c}R_{I}^{2}}{4\pi R_{T}^{4}\zeta_{o}}=\frac{\beta }{\zeta_{o}}\;,\\ \; & =\beta \gamma_{o} \end{array}$$

where we define $\beta =\frac{\sigma_{c}R_{I}^{2}}{4\pi R_{T}^{4}}$ and $\gamma_{o}=\frac{1}{\zeta_{o}}$ which we define as normalized SIR. Similarly, the resulting SIR for *K* interferers is given as,

(14) $$\begin{array}{cc} \gamma & =\frac{P_{R}}{P_{I_{1}}+P_{I_{2}}+\ldots +P_{I_{K}}},\\ \; & =\frac{\sigma_{c}}{4\pi R_{T}^{4}}\frac{1}{\sum_{n=1}^{K}\frac{\zeta_{o_{n}}}{R_{I_{n}}^{2}}}. \end{array}$$

In multiple target scenario the interference affects each target individually depending on its parameters, i.e., radar cross section (RCS) and distance of the target from the receiver. Therefore, if there are *Q* well-separated targets in the range–Doppler domain, the SIR of each target can be calculated as,

$$\begin{array}{c} \gamma_{1}=\frac{P_{R_{1}}}{P_{I}},\;\;\gamma_{2}=\frac{P_{R_{2}}}{P_{I}}\;\;\ldots \;\;\gamma_{Q}=\frac{P_{R_{Q}}}{P_{I}}, \end{array}$$

where $P_{R_{i}}$, i∈[1,Q] is the signal received from each target and $P_{I}$ is the interfering power.

### 5.5. Statistical Analysis of SIR

**Figure 6:**
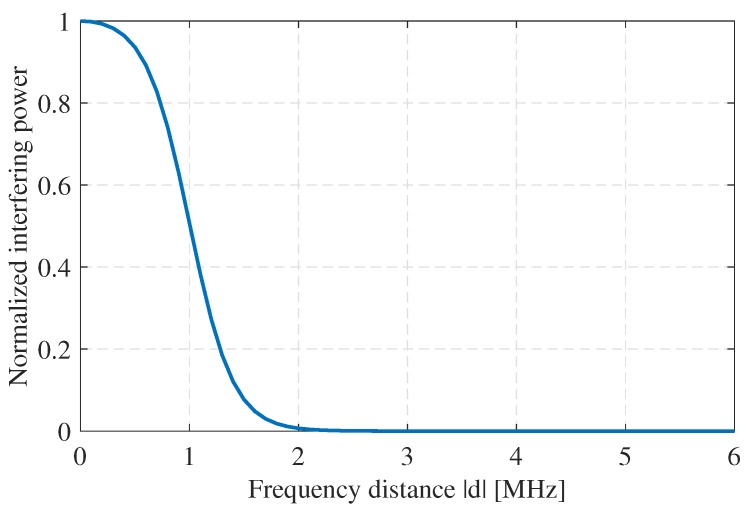
Relation between the interfering power and frequency separation between the victim radar and interfering radar at an IF/baseband filter of bandwidth 1 MHz.

The relation between the effective normalized interfering power $\zeta_{o}$ and the frequency distance between two tones is shown in Figure 6, where it is clear that $\zeta_{o}$ decreases as the frequency distance *d* between the tones increases. As we previously indicated, a PRCOS sequence will have orthogonality between its allowed phases, that is, at any given time instance two tones are separated by a frequency difference (frequency distance) denoted as *d*, where |d| ≥g. Indeed, it is better to choose a large frequency guard *g*. This will limit the maximum number of users allowed to use the sequence, $M=|\frac{N}{g}|$. So a careful allocation of frequency separation must be carried out considering both the SIR requirements and the number of users allowed.

When we consider the ordered sequences, we observe the interesting property that the distance statistics within a single column are in fact the same for all other columns (refer to Figure 4); thus, without loss of generality we can focus on the distance statistics of the first column with *M* elements.

**Figure 7:**
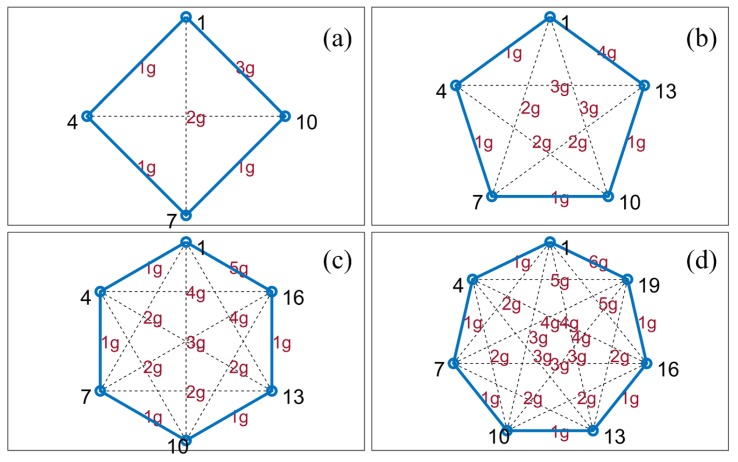
The distances between frequencies in the first column of the pseudo-random cyclic orthogonal sequence (PRCOS) with guard *g* = 3: (**a**) *M* = 4, *N* = 12 (**b**) M=5, N=15 (**c**) M=6, N=18 (**d**) M=7, N=21.

We can think of the possible interactions between these elements as a graph with *M* nodes (as shown in Figure 7) for which all nodes have equal degrees of M−1, where the label of the edge represents the distance |ng| with n∈[1,M−1]. For this study we have considered that the phases of each sequence are perfectly synchronised. In practical situations these synchronizations can be achieved with mechanisms such as GPS assisted time synchronization [46].

Accordingly, when two phases are randomly selected, the probability mass function (PMF) of the distance can be derived as follows. Consider a phase, $\phi_{j}$, is randomly chosen from M orthogonal phases with a probability of $\frac{1}{M}$, then the probability that the phase, $\phi_{i}$ selected by another radar, where i≠j will have a distance of ng is given by,

(15) $$\begin{array}{cc} P\left[d=ng\right] & =2\frac{M-n}{M(M-1)}\;:\;n\in \left[1,M-1\right]\;\forall n\in N. \end{array}$$

This can be explained by taking an example from Figure 7 with N=15 and M=5.

As depicted in Table 2, if $\phi_{1}$ is selected then the probability that the next phase has a distance of 1g is $\frac{1}{M-1}$ (refer to Figure 7). Similarly we can find the probabilities of distance between any orthogonal phases. After considering all the possible phases and their distance probabilities, we add the columns to find the probabilities of getting a distance of 1g, 2g and so on.

**Figure 8:**
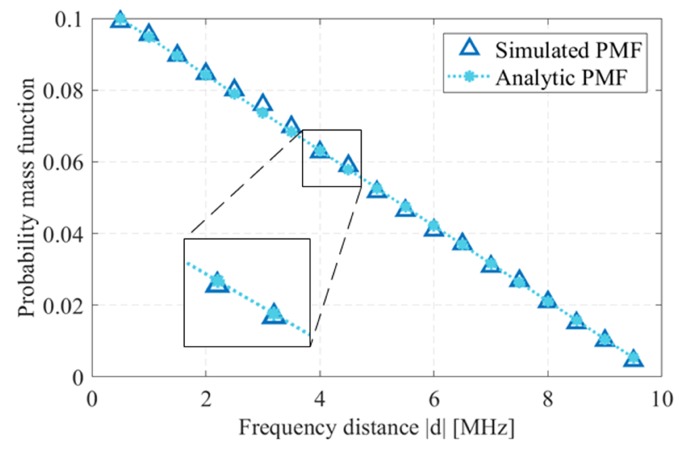
Probability mass function of frequency distance *d* with a frequency guard g=0.5 MHz.

Figure 8 shows the PMF of the frequency distance between the radars with a minimum frequency gap, *g* of 0.5 MHz

(16) $$\begin{array}{cc} P\left[d=ng\right] & =P\left[\mathrm{Choosing}\;a\;\mathrm{particular}\;\mathrm{phase}\right]\\ \; & \;\times P\left[\mathrm{Getting}\;d=ng\right]\;\forall \;n\in \left[1,M-1\right]\\ \; & =\frac{1}{M}\sum_{i=1}^{M}P_{\mathrm{when}\;\mathrm{choosing}\phi_{i}}\left[d=ng\right]\\ \; & =\frac{1}{M}\frac{2(M-n)}{\left(M-1\right)}\;,\;\forall n\in \left[1,M-1\right]. \end{array}$$

Since the normalized SIR depends on the frequency distance *d*, we can find the PMF of $\gamma_{o}$ via the transformation of random variables since $\gamma_{o}$ and *d* are related. Therefore the PMF of $\gamma_{o}$ when two orthogonal phases are randomly chosen is given by,

(17) $$\begin{array}{cc} \; & P\left[\gamma_{o}\left(d\right)=\gamma_{o}\left(ng\right)\right]=\frac{1}{M}\frac{2(M-\gamma_{o}^{-1}\left(n\right))}{\left(M-1\right)}\\ \; & =2\frac{M-\frac{C}{g}\cosh^{-1}\left(\gamma_{o}AC\sinh\left(B/C\right)-\cosh\left(B/C\right)\right)}{M(M-1)}. \end{array}$$

Thus, we obtain the expected SIR with *M* users using the PRCOS with a frequency guard of *g*, as follows,

(18) $$\begin{array}{cc} E[\gamma_{o}] & =\sum_{n=1}^{M}\gamma_{o}\left(ng\right)P\left[\gamma_{o}\left(d\right)=\gamma_{o}\left(ng\right)\right]\\ \; & =\sum_{n=1}^{M}\frac{AC\sinh\left(B/C\right)}{\cosh\left(B/C\right)+\cosh\left(ng/C\right)}\frac{2(M-n)}{M(M-1)}. \end{array}$$

In order for a radar to detect a target a minimum SIR needs to be achieved; we denote this threshold as θ. Accordingly the probability of obtaining $\gamma_{o}$ above θ is given by,

(19) $$\begin{array}{c} P_{s}=P\left(\gamma_{o}>\theta \right)=1-F_{\gamma_{o}}\left(\theta \right), \end{array}$$

where $F_{\gamma_{o}}\left(\theta \right)$ is the cumulative distribution function (CDF) of $\gamma_{o}$ and is given by,

$$\begin{array}{c} F\left[\gamma_{o}\geq \theta \right]=\sum_{i=-\infty }^{n}P\left[\gamma_{o}=\gamma_{o}\left(ig\right)\right],\;\mathrm{where}\;n=\frac{\gamma_{o}^{-1}\left(\theta \right)}{g}. \end{array}$$

**Figure 9:**
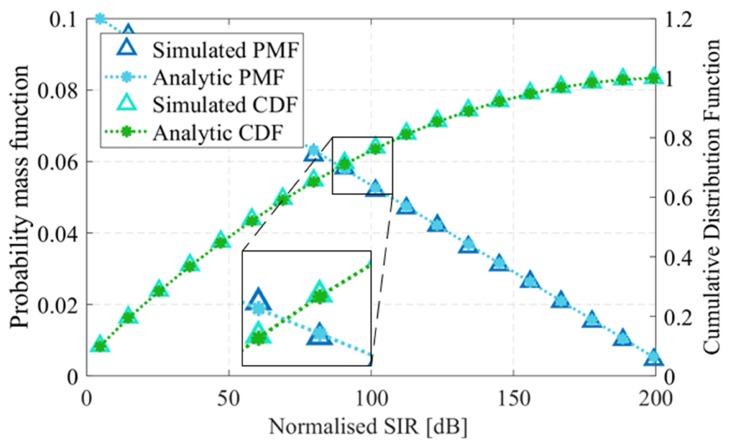
Probability mass function (PMF) and cumulative distribution function (CDF) of the normalized signal-to-interference ratio (SIR) with a frequency guard of g=0.5 MHz and N=100 tones.

Figure 9 shows the PMF and CDF of the normalized SIR for a sequence with a bandwidth, BW of 10 MHz, frequency steps Δf of 100 kHz and frequency guard *g* of 500 kHz. From Figure 9, if we consider a threshold θ of 25 dB, the probability of getting $\gamma_{o}$ above this threshold is 1 – F[θ] which gives a ranging success for 71.58% of the time, considering $\gamma_{o}$ as the normalized SIR when the received and interference power are equal at the front end of the radar.

## 6. Simulation Results

The performance of the PRCOS is analyzed by comparing the SIR with the well-known random sequence using simulations as the number of users increases for different frequency guards *g*. We generated the proposed PRCOS sequence using the algorithm explained in Section 4.2. We use a sequence of bandwidth, BW 10 MHz, with a frequency step, Δf of 100 kHz. The bandwidth for the IF/baseband LPF, *B* is taken as 400kHz in order to have a wider band compared to the frequency steps. The saturation of the front-end of the victim radar is not considered for the simulation. The frequency guard, *g*, is switched from 100 kHz to 500 kHz and the number of interferers considered is nine. In order to place the interferers we create a road strip with the first interferer at a distance $R_{I}$ of 20 m from the victim and succeeding interferers 20 m apart from each other. We place our target at a distance $R_{T}$ of 3 m from the victim radar with an RCS $\sigma_{c}$ of 100 m${}^{2}$ [15].

**Figure 10:**
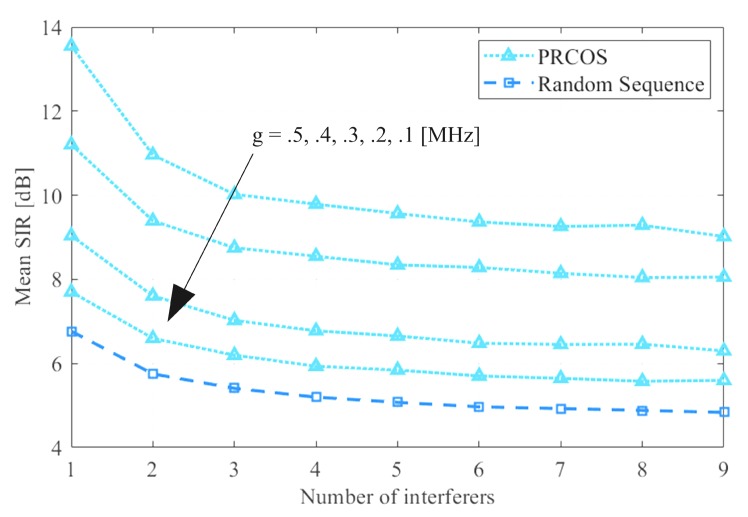
Simulation results of comparing the mean SIR for nine interferers using the PRCOS and random sequence; parameters are described in Table 4.

We first generate the PRCOS with g=100 kHz, where each interferer uses one of the generated sequences. Effective interfering power coming from interferers is calculated using Equation (12) and SIR is calculated using Equation (14). The interferers and victim interacted 1000 times to compute the mean SIR. Similar interactions are made for the sequences with g = 200, 300, 400 and 500 kHz and calculated the mean SIR. For each case of *g*, the mean SIR is computed for different cases of numbers of interferers starting from one to nine. It is obvious that, as the number of interferers increases, SIR will decrease because of the increase in the interfering power as is shown in Figure 10. In order to compare the performance of PRCOS with the well-known random stepped frequency waveform, we generate an RSFW with the same waveform parameters of PRCOS except *g* which is 0 Hz. The mean SIR is calculated for different cases of numbers of interferers from one to nine. It is evident from Figure 10 that the proposed sequence outperforms the random sequence by a significant increase in mean SIR as the frequency guard increases.

## 7. Experimental Results

**Figure 11:**
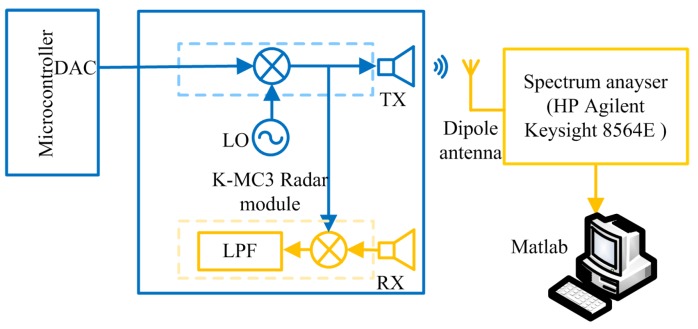
The block diagram of the experiment to verify the power spectral density (PSD) spreading.

**Figure 12:**
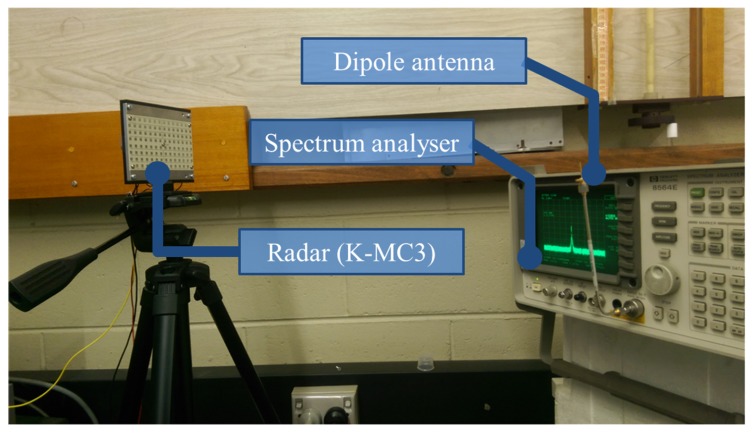
Experimental setup for measuring the PSD of pulses using a K-MC3 radar module [47].

**Figure 13:**
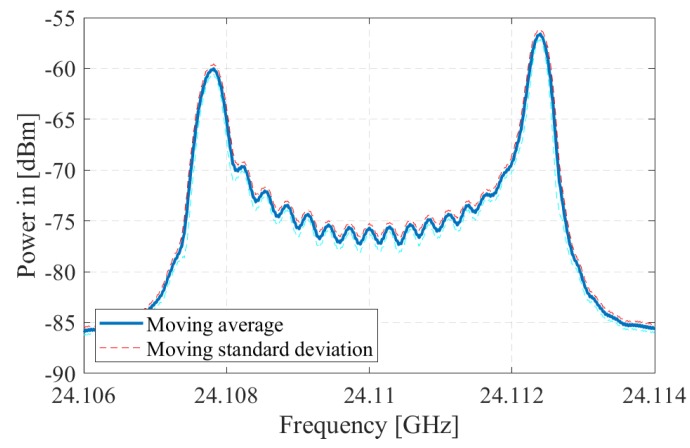
The spectrum obtained by modulating the voltage controlled oscillator (VCO) of a K-MC3 radar module with two alternating frequencies.

In order to verify the proposed model for the effective interference power, we observe the PSD spreading in a commercially available radar module, K-MC3 [47]. Since the PRCOS sequences are random frequency tones/pulses, the VCO performance of the radar module for a pulse modulation is studied by modulating the VCO using a train of pulses. We observe the RF output of the radar module by switching the VCO between two frequencies driven by the DAC output of a microcontroller. The radar module has an operating band from 24.05 GHz to 24.25 GHz with a center frequency $f_{o}$ of 24.15 GHz [47] which is regulated under the act named low interference potential devices (LIPD). Each frequency is retained for approximately 3 μs (i.e., the pulse width *T* of the DAC). The transmitted signal from the radar is captured using a dipole antenna and fed into a spectrum analyser. The experiment diagram and the actual setup are shown in Figure 11 and Figure 12, respectively. The obtained spectrum has two peaks corresponding to the two modulated frequencies, which are 24.1078 GHz and 24.1124 GHz as shown in Figure 13. The spectrum is captured over multiple times; therefore, we find the mean of the spectrum using a moving average filter with window size 180 kHz and obtain the lower and upper standard deviations using moving standard deviations.

**Figure 14:**
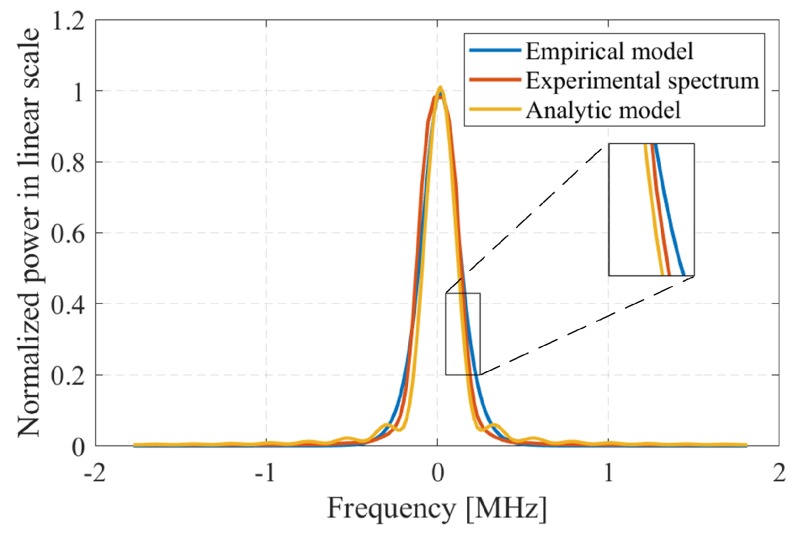
The empirical model in Equation (12) compared with the experimental results and the analytic Lorentzian model.

The PSDs of the pulses are acquired from the spectrum analyser using MATLAB^®^. We extract the spectrum of a single pulse peaked at 24.1124 GHz, which has twice the average power (due to the configured pulse width) to verify the proposed model. We find the average at each frequency sample and normalize the spectrum as shown in Figure 14. The raw data obtained from the experiment is provided online in this link [48] and the utilised parameters for the experiment are shown in Table 3.

Along with the spectrum obtained from the experiment (which is normalized), we depict the proposed empirical model and the analytic Lorentzian model obtained from the numerical analysis in Figure 14. Our empirical model fits well with experimental result as well as numerical analysis for the parameters listed in Table 4.

## 8. Conclusions

In this paper we presented an analytic framework for characterizing the mutual interference between automotive radars by modelling the phase noise and the effect of pulsed waveform on the interference. Compared to the existing studies on interference mitigation, the proposed interference model is generic and can be applied to a large class of waveforms. We introduced a novel approach in designing a family of waveform sequences for automotive radars based on pseudo-random frequency hopping that has a tractable performance. Therefore, depending on traffic densities and SIR requirements, the radars can adjust the frequency distance without a centralized control system. One of the main challenges of implementing PRCOS is that it requires a fast digital-to-analog converter (DAC) to drive/modulate the VCO; however, the modulator of existing automotive radar chipsets is based on an FMCW ramp generator. With the proposed approach using PRCOS waveforms, it is possible to predict the statistics of the radar signal-to-interference plus noise ratio based on given scenario parameters. Simulation results showed that the proposed PRCOS waveforms outperform random stepped frequency waveforms in terms of the signal-to-interference ratio by at least 7 dB. Furthermore, the presented experimental verification asserted the plausibility of the proposed analytic framework based on commercially available radar modules.

## Figures and Tables

**Table 1:** Literature review.

Ref.	Technique	Waveform
[22]	Signal processing and adaptive beam-forming	Chirp sequence
[25]	Adaptive frequency hopping	FMCW
[3]	Frequency domain	FMCW
[24]	Adaptive beam forming	Chirp sequence
[21]	Real-time signal processing	Chirp sequence
[26]	Signal processing	Chirp sequence
[27]	Coding technique	CDMA
[15,16]	Polarization and coding technique	CDMA
[13]	Time domain, frequency domain and coding technique	FMCW, modulated pulses
[17]	Time domain	Pulse waveform
[28,29]	Frequency domain	Stepped frequency waveform

**Table 2:** *N* = 15, *M* = 5.

	P[d=ng]	P[d=1g]	P[d=2g]	P[d=3g]	P[d=4g]
Phases Φ					
$\phi_{1}$		$\frac{1}{M-1}$	$\frac{1}{M-1}$	$\frac{1}{M-1}$	$\frac{1}{M-1}$
$\phi_{2}$		$\frac{2}{M-1}$	$\frac{1}{M-1}$	$\frac{1}{M-1}$	0
$\phi_{3}$		$\frac{2}{M-1}$	$\frac{2}{M-1}$	0	0
$\phi_{4}$		$\frac{2}{M-1}$	$\frac{1}{M-1}$	$\frac{1}{M-1}$	0
$\phi_{5}$		$\frac{1}{M-1}$	$\frac{1}{M-1}$	$\frac{1}{M-1}$	$\frac{1}{M-1}$

**Table 3:** Parameters of the experiment.

Parameter	Value
Spectrum analyser start frequency	24.104 GHz
Spectrum analyser stop frequency	24.115 GHz
Spectrum analyser resolution bandwidth	10 kHz
Spectrum analyser video bandwidth	1 kHz
Radar center frequency of pulse 1	24.1978 GHz
Radar center frequency of pulse 2	24.1124 GHz
Moving average window	180 kHz

**Table 4:** Notations and symbols.

Parameter	Symbol	Value
Received power	$P_{R}$	-
Transmitted power	$P_{o}$	-
Radar cross section	$\sigma_{c}$	100 $m^{2}$
Effective aperture	$A_{e}$	-
Distance to target	$R_{T}$	3 m
Wavelength	λ	1.24 cm
Gain of the antenna	*G*	-
Interfering power	$P_{I}$	-
Distance to interferer	$R_{I}$	20–180 m
Signal to interfering ratio	γ	-
Effective normalized interfering power	$\zeta_{o}$	-
Noise variance	$\sigma_{n}$	-
Phase noise power spectral density	£(f)	-
Scalar constant of phase noise	α	-
Center frequency	$f_{o}$	24.15 GHz
Pulse width	*T*	3 μs
Pulse amplitude	*E*	-
Frequency step	Δf	100 kHz
Bandwidth of RF	BW	10 MHz
Frequency guard	*g*	0.1–0.5 MHz
Frequency distance	*d*	-
Relative starting point of the sequence	ϕ	-
Empirical model amplitude level	*A*	0.24
Empirical model spread of the signal	*C*	200 kHz
Bandwidth of the IF/Baseband LPF	*B*	400 kHz
Number of frequency tones	*N*	100
Normalized signal to interference ratio	$\gamma_{o}$	-
Number of targets	*Q*	1
Number of users	*M*	1
SIR Threshold	θ	25 dB
